# Ruptured Perineum

**Published:** 1875-03

**Authors:** Robert Battey

**Affiliations:** Atlanta, Ga.


### RUPTURED PERINEUM.

By ROBERT BATTEY, M.D., Atlanta, Ga.

*Read before the Atlanta Academy of Medicine.*

Case 1.—Mrs. T., aged 24, married, mother of two children, a lady from Texas, consulted me for an injury which she had received in her last confinement, and which, she informed me, weighed much upon her mind. An exploration of the case disclosed an incomplete rupture of the perineum; it was not extensive; the uterus was healthy and in situ; the menstrual function normal and painless; no complaint of back-ache; indeed, the general health was excellent. I advised the lady of her condition, and counseled her to dismiss the matter from her mind and content herself to let well-enough alone. She was evidently deeply concerned, and could not accept my advice, for she rejoined, with earnestness in her manner, "Doctor, *something must be done.*" A further probing of the case developed the facts that she had suffered nothing herself from the injury; indeed, was not aware of the existence of any injury, until she was told by her husband that "her last labor had ruined her." It was the happiness, then, of her husband, coupled with the apprehension of alienation of his affections, which so distressed her, and not any physical discomfort upon her own part. In this new view of the case there could be no room for hesitation. She was therefore subjected to operation. The ruptured surfaces were pared, or rather denuded, with a bistoury and forceps, and brought together with three silver wires and secured beneath my combined splint and compress, as described in the *London Lancet* of 1860. The apparatus was allowed to remain for ten days, the bowels acting daily, and the patient permitted to move about her room at her own option, when the parts were found to be firmly and smoothly healed, to her infinite joy and satisfaction.

Case 2.—Mrs. M., Chattooga county, Ga., aged 20, married. Lost one child in a very protracted and difficult labor requiring instrumental delivery, which was attended by laceration of the perineum and sphincter muscle. Solid motions were retained with ease, but liquids and gas could not be controlled. She had a very slow getting up from her labor, and was extremely anæmic when she presented herself for operation, six months subsequent to her confinement. This case was operated precisely as the last, with exception that a larger number of sutures was required. The patient was confined to bed and the bowels constipated. On the ninth day the sutures were removed, and the union proved to be perfect, excepting a small point at the sphincter. A single wire suture was passed to support the sphincter, and allowed to remain for weeks after all was soundly healed. In this case, as in the former one, the perineum was smoothly and beautifully moulded by the leaden splint, up against which it was firmly and evenly drawn by the ox-bow form of the wire sutures.

Case 3.—Mrs. L., of Fulton county, Georgia, aged 20, married, ruptured in her first labor. She was in experienced and skilful hands, and yet, as not unfrequently happens, the existence of the laceration was unknown until the following day. The rent implicated the superficial portion of the sphincter muscle, but there was remaining good power of control over the rectal contents. She was seen upon the fourth day, but, notwithstanding the lapse of time, it was deemed best to bring the surfaces together with deeply lodged sutures of silver wire. The wires were allowed to remain for weeks, and until it was evident that they were no longer giving useful support. The parts healed slowly by granulation, until eventually a satisfactory cure was obtained, under the occasional stimulation of the nitrate of silver.

Case 4.—Mrs. T., of Lee county, Georgia, aged 34, married, mother of eight children, emaciated and feeble from long-standing chronic diarrhœa. In her first labor, sixteen years ago, she sustained injury by extensive laceration of the perineum, and she thinks the rent has been somewhat enlarged by her subsequent deliveries. From the first she was entirely deprived of all power of control over the intestinal contents, and chronic diarrhœa was the consequence. She has been repeatedly advised by her physicians that it would be entirely useless to attempt any surgical measure, in the hope of a restoration of the normal type of the parts. Upon inspection I found, perhaps, the most extensive laceration of the perineum, sphincter and recto-vaginal wall which it has ever been my fortune to encounter. The sphincter muscle, by its long disuse, had contracted itself into a nodular mass at the termination of the posterior rectal wall; it required an effort of surgical expectation to picture it again in its proper annular form, surrounding the anus and performing its natural functions. Above the sphincter the rent extended an inch and a half, or more, up the rectum, and the posterior rectal wall was prolapsed and projected forward, presenting a vascular ruby tumor, just within the genital fissure.

With the assistance of Drs. Westmoreland, Holmes and Kendrick, the patient was etherized, and the surfaces pared with scissors throughout. The rent in the recto-vaginal septum was brought together, requiring five points of silver wire suture, and the perineum united by four wire sutures passing through perforated metallic quills on either side and secured by shot. In the after-treatment the diarrhœa was controlled by opiates, coupled with an astringent pill. The urine was drawn at intervals for two days, when it was found that the patient could pass it more comfortably into a tin cup placed below the labium as she lay upon her side. A vesical irritation supervened upon the fourth day, and proved a source of some distress, the urine often passing involuntarily. On the fourth day the perineal sutures were removed, and the union proved to be complete. The metallic quills were burying themselves rather deeply in the tissues, so that they could not well be allowed to remain longer. On the ninth day several spontaneous evacuations of the bowels of rather soft consistency occurred, and all seemed tight and firm. Gas passed naturally through the sphincter for several days, and was under perfect control. Upon the tenth day the sutures within the vagina were removed, and all seemed perfect throughout, but after an evacuation occurring some hours subsequently, gas was observed to pass through the rectal wall above the sphincter and there was some oozing into the vagina of liquid from the rectum. Two days subsequently the little opening was granulating, and liquid matters were well retained, but gas still found its way through. The union of the sphincter muscle is perfect, and its physiological action entirely normal. On the eighteenth day she returned home, with good control of her rectal functions, and reports herself on the 3d of February entirely well.

REMARKS.—The foregoing cases are selected from my notebook for the illustration of different methods of treatment of ruptured perineum. Undoubtedly this lesion of tissue may be successfully repaired by a variety of methods, and the surgeon may fairly be left to his individual choice in the selection for any given case. For my own method, by the leaden splint and compress, I am not disposed to claim more than that it secures here, as in hare-lip and vesico-vaginal fistula, the smoothest and handsomest result. Although this is of less consequence in the perineum than in the lip and vesico-vaginal septum, still it is everywhere desirable. The ox-bow form of my suture, like the simple wire loop of Sims, may be left in the tissues for any necessary period without difficulty. The action of the compress is not alone to smoothly mould the cicatrix, but also to condense the tissues and prevent the insinuation of vaginal and rectal secretions between the pared surfaces, and it likewise acts as a shield to prevent the urine from coming in contact with the approximated edges. In this operation too much stress cannot be laid upon the importance of bringing firmly together the divided ends of the sphincter muscle, as has been so clearly pointed out by Emmet, Thomas, Agnew and others.

When ought a ruptured perineum to be operated? Can there be a better time than that which immediately follows the occurrence of the rupture? It is well known that partial rupture of the perineum in labor is a very common occurence, much more common than is supposed by those who are not specially watching for it. It is as well known that ruptures of a considerable extent, which do not involve the complete division of the sphincter muscle, not unfrequently make excellent recoveries by granulation. Immediately after labor the perineum is so much benumbed and the patient so inured to pain as to bear the passage of the needle without the necessity of anæsthesia; the patient then bears exposure of the vulva with less shock to her feminine delicacy than at a subsequent period; and the surfaces are fresh and oozing. What more could be desired than to bring the raw surfaces smoothly and firmly in contact, and protect them with the leaden splint and compress, and this to remain until all is firmly healed?

